# The Impact of Coronary Ischemia Assessment on Outcomes in Those With Scar‐Dependent Ventricular Tachycardia

**DOI:** 10.1111/jce.16495

**Published:** 2024-11-15

**Authors:** Michael C. Waight, Davide Fabbricatore, Elijah R. Behr, Manav Sohal, Anthony C. Li, Magdi M. Saba

**Affiliations:** ^1^ City St George's University of London London UK; ^2^ St George's University Hospitals NHS Foundation Trust London UK; ^3^ Cleveland Clinic of London London UK

**Keywords:** coronary angiogram, ischemia assessment, ventricular tachycardia

## Abstract

**Background:**

Guidance and outcomes of coronary ischemia assessment (IA) in those with structural heart disease (SHD), presenting with monomorphic ventricular tachycardia (MMVT) is unclear.

**Objectives:**

To assess the impact of IA on arrhythmic and non‐arrhythmic outcomes in those with SHD.

**Methods:**

Patients presenting with MMVT over a 6‐year period to a tertiary center were retrospectively analyzed. Propensity score‐matched analysis was performed comparing those undergoing IA to those who did not. The primary endpoint was a composite of VT recurrence, appropriate ICD therapy, heart failure hospitalization, and death. Secondary analysis of the individual components of the composite was performed. Kaplan–Meier, univariate and multivariate analysis was performed to compare the two groups and derive predictors of poor outcomes.

**Results:**

Two hundred and seventeen patients (57.6% ICM) were analyzed. 55.8% underwent IA. Following propensity score‐matching, 120 patients remained. At 12 months, freedom from the primary endpoint was 68.3% of those undergoing IA versus 43.3% who did not, *p* < 0.001, multivariate HR 0.56 (0.34–0.92). This was driven by a reduction in all‐cause mortality, with a 12‐month survival of 98.3% in those undergoing IA versus 86.5% in those not undergoing IA (*p* < 0.01). Coronary intervention was associated with a significantly higher event‐free 12‐month survival compared to those who did not undergo intervention (82.4% vs 51.5%, respectively, *p* = 0.01).

**Conclusions:**

Patients with SHD presenting MMVT who undergo an IA have significantly improved freedom from VT recurrence, appropriate ICD therapies, HF hospitalization, and death compared to those who do not, driven by a reduction in mortality.

## Graphical abstract

**Central illustration:** In those presenting With monomorphic VT, undergoing coronary ischemia assessment was associated With improved 12‐month event‐free survival from the primary endpoint of VT recurrence, ICD therapy, heart failure hospitalization, and death.

**Abbreviations**
CAD: coronary artery disease.
CKD: chronic kidney disease.
CMR: cardiac magnetic resonance.
IA: ischemia assessment.
ICD: implantable cardioverter‐defibrillator.
SCD: sudden cardiac death.
SHD: structural heart disease

## 1 Introduction

Ischemic heart disease is the commonest etiology leading to sudden cardiac death (SCD) worldwide [1] with ventricular arrhythmias being the responsible cause in a significant proportion of cases [2]. Increasing burden of coronary artery disease (CAD) has been shown to be a poor prognostic marker in both ischemic (ICM) and nonischemic cardiomyopathy (NICM) [3, 4] and current guidelines on the assessment of those surviving SCD strongly advocate the use of coronary angiography as part of the diagnostic evaluation. However, for those presenting with monomorphic VT (MMVT), guidance on ischemia assessment (IA) is less clear [5, 6] The impact of coronary imaging and intervention in those with structural heart disease (SHD) on future arrhythmic and nonarrhythmic outcomes requires further evaluation. Furthermore, local practice is variable. We aim to assess real‐world outcome data in patients with SHD presenting with MMVT, comparing those who undergo a coronary IA to those who do not and try to derive predictors of poor future outcomes.

## 2 Methods

### 2.1 Study Setting

St George's Hospital is a high‐volume tertiary referral center for patients with ventricular arrhythmias. From a local registry, consecutive patients admitted to St George's cardiology department between 01/01/2016 and 24/03/2022 with a diagnosis of MMVT were identified. Patients were identified through ICD‐10 classification coding from their electronic patient record. Inclusion criteria were any patient admitted with a primary diagnosis of monomorphic VT during their hospital stay. Exclusion criteria were age < 18 years, those without evidence of SHD on diagnostic workup (normal heart VT), VT in the context of acute myocardial infarction and those with VT secondary to exogenous causes such as drugs and transient metabolic derangements.

### 2.2 Data Collection

Data on baseline demographics, length of stay, medication usage, left ventricular ejection fraction (LVEF) on echocardiography or cardiac magnetic resonance (CMR), troponin measurement, underlying etiology (ICM vs NICM determined by contemporaneous or historic records and investigations) and presence or absence of IA were collected. Those with mixed ICM/NICM were categorized into one or other group based on the predominant cause based on coronary angiography and CMR/echo. For a diagnosis of predominant NICM, the degree of left ventricular dysfunction needed to be out of proportion to the extent of coronary disease. All forms of IA (invasive and noninvasive) were considered and were counted if the assessment took place during, or within the 12 months preceding, the index admission. For those who underwent coronary angiography (invasive or CT), severity of coronary disease was assessed using the CAD‐RADS system, classifying the lesions into none (0%), mild (1%–49%), moderate (50%–69%) and severe (> 70%). For those with previous coronary artery bypass grafting (CABG), the severity of coronary disease was based on the grafts (where present), rather than native vessels. Data on coronary intervention (percutaneous or surgical) were collected. IA and intervention were performed at the senior physician's discretion. A patient was considered to have undergone VT ablation if this occurred within the 12 months following their admission with VT.

### 2.3 Endpoints

Patients were followed up until 01/05/2023. The primary endpoint was a composite of recurrent ventricular arrhythmia, heart failure hospitalization, and all‐cause mortality. Recurrence of ventricular arrhythmia was defined as documented sustained VT/VF or any appropriate ICD therapy. Duration of follow‐up was calculated from the day of first admission with MMVT to the day the primary endpoint occurred or last patient contact. Event‐free survival in patients with follow‐up to 12 months was assessed in those with and without the occurrence of the primary endpoint. Secondary endpoint analysis included recurrence of ventricular arrhythmia/appropriate ICD therapy, heart failure hospitalization, and all‐cause mortality.

### 2.4 Statistical Analysis

Continuous data are expressed as mean ± standard deviation or median with interquartile range. Categorical data are expressed as relative counts and percentages. Data were tested for normality using the Kolmogorov‐Smirnov test. Means of continuous data were compared using independent samples *t*‐test after controlling for equality of variance with Levene's test. Means of non‐parametric variables were compared using the Mann–Whitney U test. Categorical data was compared using Fisher's exact test or chi‐square test as appropriate.

Propensity score (PS) matching was carried out to control for differences in baseline variables including age, gender, LVEF, etiology (ICM or NICM), diabetes, atrial fibrillation, hypertension, chronic kidney disease (CKD), Antiarrhythmic drug use (including beta blockers, class I and class III agents), heart failure medication use and VT ablation status. Case‐control matching was performed in a 1:1 ratio using the nearest‐neighbor method with a caliper width of 0.2 standard deviations. Following PS matching, the between group standardized mean difference was verified for all baseline variables.

Following PS matching, Kaplan–Meier curves were constructed to demonstrate cumulative survival and tested for significance using logrank tests. Subgroup analysis was conducted for those with ICM and NICM. Univariate logistic regression analysis was performed to analyze factors associated with the primary endpoint and are expressed as odds ratio (OR) and 95% confidence interval (CI). Factors which were significant on univariate analysis were entered into a multivariate logistic regression analysis.

All tests were two‐sided and a *p*‐value of < 0.05 was taken to indicate statistical significance. Statistical analysis was carried out with SPSS version 30 (IBM Corp, Armonk, NY, USA) and R version 4.3.1 (R Foundation for Statistical Computing, Vienna, Austria).

This study was granted ethical approval by the local review board and is in compliance with the Declaration of Helsinki. Patients gave their informed consent to participate.

## 3 Results

### 3.1 Full Cohort Results

#### 3.1.1 Patient Characteristics

Between January 1, 2016 and March 24, 2022, 290 consecutive patients were identified as presenting with MMVT. Forty‐nine individuals were excluded due to a lack of adequate follow‐up data. A further 24 patients were excluded due to lack of underlying SHD, leaving 217 patients in the final analysis (82.9% male, mean age 67.8 (± 14.9) years). Median follow‐up was 1093 (622–1688) days. Mean LVEF was 37.3% (± 13.4%). 125/217 (57.6%) had an ischemic cardiomyopathy as the predominant etiology, whereas 92/217 (42.4%) had a NICM. The NICM cohort comprised 56.5% dilated cardiomyopathy (DCM), 19.6% arrhythmogenic cardiomyopathy (ACM), 9.8% infiltrative, 5.4% hypertrophic cardiomyopathy (HCM), 5.4% myocarditis and 3.3% valvular heart disease. 98/217 (45.2%) had an ICD in situ on first presentation. 17/217 (7.8%) presented with VT storm. Compared to those with NICM, patients with ICM were older (72.2 vs 61.8 years, *p* < 0.001), more likely to be male (88% vs 76%, *p* < 0.001), with a greater prevalence of hypertension (44.0% vs 29.3%, *p* < 0.001), diabetes (36.0% vs 22.8%, *p* < 0.001) and chronic kidney disease (42.4% vs 25.0%, *p* = 0.008). Full demographics of the full cohort are listed in Table 1.

**Table 1:** Demographics of the full cohort.

Demographic	ICM (125)	NICM (92)	Total (217)
Male gender	110 (88.0%)	70 (76.1%)	180 (82.9%)
Mean age	72.2 (± 9.9)	61.8 (± 18.1)	67.8 (± 14.9)
Hypertension	55 (44.0%)	27 (29.3%)	82 (37.8%)
Diabetes	45 (36.0%)	21 (22.8%)	66 (30.4%)
Atrial fibrillation	41 (32.8%)	31 (33.7%)	72 (33.2%)
Chronic kidney disease	53 (42.4%)	23 (25.0%)	76 (35.0%)
Baseline EF (%)	35.2 (± 12.4)	40.1 (± 14.3)	37.3%
B‐blocker	99 (79.2%)	65 (70.7%)	164 (75.6%)
Amiodarone	9 (7.2%)	9 (9.8%)	18 (8.3%)
Other AAD	3 (2.4%)	2 (2.2%)	5 (2.3%)
Antithrombotic	109 (87.2%)	54 (58.7%)	163 (75.1%)
Statin	103 (82.4%)	41 (44.6%)	144 (66.4%)
ACE‐I/ARB	91 (72.8%)	55 (59.8%)	146 (67.3%)
MRA	52 (41.6%)	26 (28.3%)	78 (35.9%)
ARNI/SGLT‐2‐I	9 (7.2%)	5 (5.4%)	14 (6.5%)
Length of stay (days)	11.5 (± 14.0)	9.5 (± 7.5)	11.5 (± 14.0)
ICD on admission	53 (42.4%)	45 (48.9%)	98 (45.2%)
Mean number of episodes of sustained VT	4.3 (± 10.5)	5.6 (± 11.3)	4.8 (± 10.8)
VT storm	11 (8.8%)	6 (6.5%)	17 (7.8%)

Abbreviations: AAD = antiarrhythmic drug; ARNI = angiotensin receptor‐neprilysin Inhibitor; SGLT‐2‐I = sodium‐glucose cotransporter‐2 inhibitor.

#### 3.1.2 Investigations, Intervention and Management of Arrhythmia

121/217 (55.8%) patients presenting with VT underwent an IA. The assessment was with a coronary angiogram in 97.5% of cases and this occurred during the index admission in 98.3% of cases. Cardiac MRI was performed in 73/217 (33.6%) of cases. Troponin T was recorded in 210/217 (96.8%). A troponin rise of > 50% from baseline was recorded in 115/210 (54.8%) of cases. Of the 121 individuals who underwent an IA, coronary disease was assessed as none or mild in 41/121 (33.9%), moderate in 17/121 (14.0%), and severe in 64/121 (52.9%). Coronary intervention was performed in 27/121 (22.3%) of individuals who underwent an IA, all of whom exhibited severe coronary disease on angiography. This comprised percutaneous coronary intervention (PCI) in 22 (81.5%) and CABG in 5/27 (18.5%). 7/27 (25.9%) patients who underwent revascularisation had a physiological IA (pressure wire study). Of the 37 patients who had severe coronary disease who did not undergo intervention, there was a documented reason for this in 28 (75.7%), including lack of viability on CMR, negative pressure wire assessment, chronic total occlusion with absence of chest pain and technical feasibility.

182/217 (83.9%) patients had escalation of their antiarrhythmic drug therapy. 25/217 (11.5%) underwent emergent inpatient VT ablation. 36/217 (16.5%) underwent deferred VT ablation during the following 12 months. IA was performed more frequently in those with ICM versus NICM (81/125 [64.8%] vs 40/92 [43.5%], *p* = 0.02). Coronary disease was more likely to be severe and less likely to be absent (58/81 [71.6%] vs 6/40 [15.0%], *p* < 0.001 and 2/81 [2.5%] vs 21/40 [52.5%], *p* < 0.001 respectively. Those with ICM were more likely to undergo coronary intervention (26/81 [32.1%] vs 1/40 [2.5%], *p* < 0.001). Those with ICM were less likely to undergo VT ablation compared to NICM (28/125 [22.4%] vs 33/92 [35.9%], *p* = 0.03). There was no significant difference between the ICM and NICM with regard to frequency of cardiac MRI, measurement of troponin T, mode of revascularisation or antiarrhythmic drug escalation (Table 2).

**Table 2:** Investigations, intervention, and treatment of arrhythmia in the full cohort and in ICM versus NICM populations.

Evaluation	Total (217)	ICM (125)	NICM (92)	*p*‐value (ICM vs NICM)
Ischemia assessment performed	121 (55.8%)	81 (64.8%)	40 (43.5%)	0.02
Cardiac MRI performed	73 (33.6%)	59 (47.2%)	41 (44.6%)	0.70
Troponin T measured	210 (96.8%)	121 (96.8%)	89 (96.7%)	0.98
Coronary disease severity	*n* = 121	*n* = 81	*n* = 40	
None	27 (22.3%)	2 (2.5%)	21 (52.5%)	< 0.001
Mild	14 (11.5%)	10 (12.3%)	8 (20.0%)	0.27
Moderate	16 (13.2%)	11 (13.6%)	5 (12.5%)	0.87
Severe	64 (52.9%)	58 (71.6%)	6 (15.0%)	< 0.001
Coronary intervention	27 (22.3%)	26 (32.1%)	1 (2.5%)	< 0.001
PCI (*n* = 27)	22 (81.5%)	21 (80.8%)	1 (100%)	0.64
CABG (*n* = 27)	5 (18.5%)	5 (19.2%)	0	0.64
Antiarrhythmic drug escalation	181 (83.4%)	109 (87.2%)	72 (78.3%)	0.10
VT ablation	61 (28.1%)	28 (22.4%)	33 (35.9%)	0.03
Emergent ablation	25 (11.5%)	11 (8.8%)	14 (15.2%)	0.20
Elective ablation	36 (16.6%)	17 (13.6%)	19 (20.7%)	0.12

### 3.2 Propensity‐Matched Cohort Results

#### 3.2.1 Propensity Score‐Matched Demographics

Following PS‐matching for those who did and did not undergo an IA, 120 patients remained for analysis. There were no significant differences in the demographics or etiology between the two groups. All further analysis was performed on the PS‐matched cohorts. Demographics of this cohort are shown in Table S1.

#### 3.2.2 Predictors of Recurrence of VT, Mortality, and Heart Failure Hospitalization

**Figure 1:**
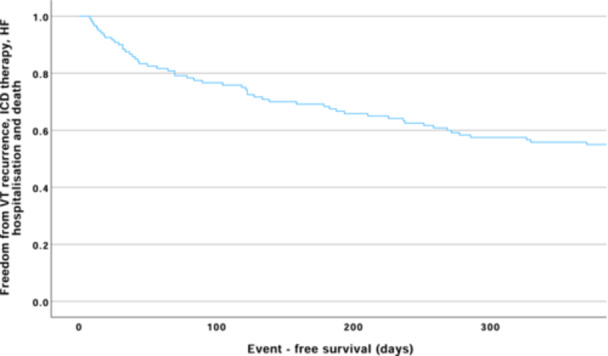
Kaplan–Meier survival curve for patients presenting with monomorphic VT with structural heart disease. The endpoint was 12‐month freedom from VT recurrence, ICD therapies, heart failure hospitalization, and death. Freedom from the primary endpoint at 12 months was 55.8% (± 4.5%).

During the follow‐up period, the primary endpoint was seen in 77/120 (64.2%) of patients. The median time from admission to reach the primary endpoint was 564 days (IQR 272–911 days). Freedom from the primary endpoint at 12 months was 55.8% (± 4.5%) (Figure 1, central illustration 1).

**Central Illustration 1:**
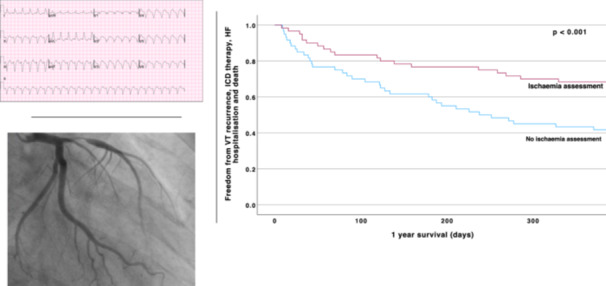
In those presenting with monomorphic VT, undergoing coronary ischaemia assessment was associated with improved 12‐month event‐free survival from the primary endpoint of VT recurrence, ICD therapy, heart failure hospitalisation and death.

**Figure 2:**
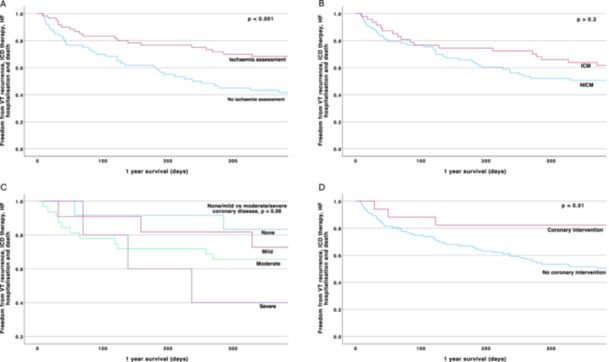
Kaplan–Meier survival curves demonstrating 12‐month event‐free survival from VT recurrence, ICD therapy, heart failure hospitalization and death. (A) Comparison of those with and without an ischemia assessment shows improved event‐free survival of those undergoing ischemia assessment. (B) Comparison of ischemic versus nonischaemic cardiomyopathy shows no significant difference in outcomes at 12 months. (C) Comparison of none, mild, moderate, or severe coronary disease shows a trend towards worse outcomes with more severe coronary disease. (D) Comparison of coronary intervention versus no coronary intervention shows improved event‐free survival in those undergoing intervention.

At 12 months, freedom from the primary endpoint was significantly higher in those who underwent an IA compared to those who did not (68.3% vs 43.3%, *p* < 0.001). There was no significant difference in adverse events when comparing ICM to NICM patients, with freedom from the primary endpoint at 12 months in 50.7% of those with ICM versus 63.8% of those with NICM (*p* = 0.2). A greater severity of coronary disease was associated with a trend towards reduced event‐free 12‐month survival (none—83.3%, mild—72.7%, moderate—65.6% and severe 40.0%, which did not reach statistical significance (*p* = 0.06 for none/mild vs moderate/severe). Coronary intervention was associated with a significantly higher event‐free 12‐month survival versus those who did not undergo coronary intervention (82.4% vs 51.5%, respectively, *p* = 0.01) (Figure 2).

Those who underwent IA were significantly more likely to undergo adjustments to heart failure and cardiovascular risk‐reducing medications. Commencement or escalation of antithrombotic therapy was observed in 20/60 (33.3%) of those who underwent IA versus 8/60 (13.3%) of those who did not undergo IA (*p* = 0.02). Escalation of heart failure medication occurred in 41/60 (68.3%) of those who did versus 27/60 (45.0%) of those who did not undergo IA (*p* = 0.02).

The proportion of patients who had optimization of anti‐arrythmic drug therapy did not differ significantly between those who did and those who did not undergo IA (51/60 [85.0%] versus 47/60 [78.3%], *p* = 0.48). Coronary revascularisation was only performed in those who underwent IA (17/60 [28.3%] versus 0/60 (0%), *p* = < 0.001). Table 3.

**Table 3:** Comparison of interventions received by those undergoing versus not undergoing ischemia assessment within the propensity‐matched cohort.

Intervention	No Ischemia assessment (*n* = 60)	Ischemia assessment (*n* = 60)	*p*‐value
Commenced/increased beta blocker	25 (41.7%)	32 (53.3%)	0.27
Commenced/increased amiodarone	24 (40.0%)	25 (41.7%)	1.0
Commenced/increased other AAD	11 (18.3%)	4 (6.7%)	0.10
Escalation of AAD	47 (78.3%)	51 (85.0%)	0.48
Commenced/increased antithrombotic	8 (13.3%)	20 (33.3%)	0.02
Commenced/increased statin	4 (6.7%)	9 (15.0%)	0.24
Commenced/increased ACE‐I/ARB	3 (5.0%)	15 (25.0%)	0.004
Commenced/increased MRA	2 (3.3%)	13 (21.7%)	0.004
Commenced/increased ARNI/SGLT‐2‐I	2 (3.3%)	1 (1.7%)	1.0
Escalation of heart failure therapy	27 (45.0%)	41 (68.3%)	0.02
ICD/CRT at discharge	55 (91.7%)	54 (90.0%)	0.75
Revascularisation performed	0 (0%)	17 (28.3%)	< 0.001
VT ablation	15 (25%)	15 (25%)	1.0

Abbreviations: AAD = anti‐arrhythmicantiarrhythmic drug; ARNI = angiotensin receptor‐neprilysin Inhibitor; SGLT‐2‐I = sodium‐glucose cotransporter‐2 inhibitor.

On univariate analysis, factors which were associated with the primary endpoint included male gender (HR 2.45 [1.18–5.11], *p* = 0.02), atrial fibrillation (HR 1.82 [1.15–2.88], *p* = 0.01), CKD (HR 1.89 [1.20–2.98], *p* < 0.001), beta blocker therapy (HR 2.85 [1.36–5.93], *p* 0.02), IA (HR 0.43 [0.27–0.69], *p* < 0.001) and coronary intervention (HR 0.36 [0.16–0.83], *p* = .02). On multivariate analysis, only IA remained significant (HR 0.61 [0.37–0.98], *p* = 0.04). (Table 4).

**Table 4:** Univariate and multivariate analysis of factors in the propensity‐matched cohort (*n* = 120) associated with the primary endpoint.

Factor	Univariate analysis			Multivariate analysis		
	Hazard ratio	CI	*p*‐value	Hazard ratio	CI	*p*‐value
Age > 65	1.43	0.87–2.37	0.16			
Male gender	2.45	1.18–5.11	0.02	2.03	0.96–4.30	0.07
Hypertension	1.04	0.65–1.66	0.88			
Diabetes	1.12	0.68–1.83	0.66			
Atrial fibrillation	1.82	1.15–2.88	0.01	1.19	0.73–1.94	0.48
Chronic kidney disease	1.89	1.20–2.98	< 0001	1.64	1.03–2.63	0.04
EF < 40%	1.48	0.93–2.36	0.10			
Discharged on beta blocker	2.08	0.65–6.60	0.22			
Discharged on amiodarone	1.30	0.83–2.04	0.26			
Escalation of AAD therapy	0.89	0.52–1.55	0.69			
Discharged on antithrombotic	0.96	0.50–1.81	0.89			
Discharged on statin	1.17	0.70–1.97	0.54			
Discharged on ACE‐I/ARB	1.64	0.92–2.94	0.09			
Discharged on MRA	0.93	0.59–1.45	0.74			
Discharged on ARNI/SGLT‐2‐I	1.44	0.53–4.00	0.48			
Escalation of heart failure therapy	0.73	0.47–1.15	0.17			
Length of stay	0.99	0.96–1.01	0.26			
Ischemia assessment	0.43	0.27–0.69	< 0.001	0.56	0.34–0.92	0.02
Moderate or severe coronary disease	2.00	0.94–4.24	0.07			
Coronary intervention	0.36	0.16–0.83	0.02	0.54	0.22–1.32	0.17
VT ablation	0.55	0.25–1.20	0.14			
Peak troponin T	1	1.00–1.00	1.00			
Scar on CMR	1.17	0.27–5.00	0.84			

Abbreviations: AAD = anti‐arrhythmicantiarrhythmic drug; ARNI = angiotensin receptor‐neprilysin Inhibitor; SGLT‐2‐I = sodium‐glucose cotransporter‐2 inhibitor.

#### 3.2.3 Secondary Endpoints

**Figure 3:**
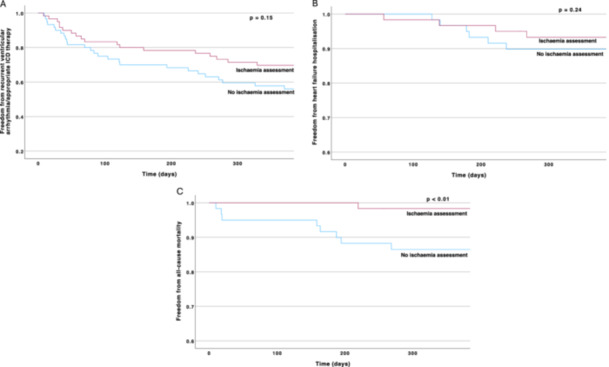
Kaplan–Meier survival curves demonstrating secondary outcomes at 12 months, stratified by presence of an ischemia assessment. (A) Recurrent ventricular arrhythmia/appropriate ICD therapy was similar between those who did and did not undergo an ischemia assessment. (B) Heart failure hospitalization was also similar between the two groups. (C) All‐cause mortality was significantly lower in the ischemia assessment group (98.3%) versus those not undergoing ischemia assessment (86.5%) (*p* < 0.01).

In secondary endpoint analysis of the PS‐matched cohort, median time to recurrent VA/appropriate ICD therapy was 967 days (IQR 518–1416 days), median time to HF hospitalization was 268 days (IQR 170–680 days), median time to all‐cause mortality was 139 days (IQR 368–1524 days). At 12 months, IA was not associated with a reduced rate of recurrent VA/appropriate ICD therapy or heart failure hospitalization. However, IA was associated with reduced mortality with a 12‐month survival of 98.3% in those undergoing IA versus 86.5% in those not undergoing IA (*p* < 0.01) (Figure 3).

#### 3.2.4 Subgroup Analysis of ICM and NICM Patients

**Figure 4:**
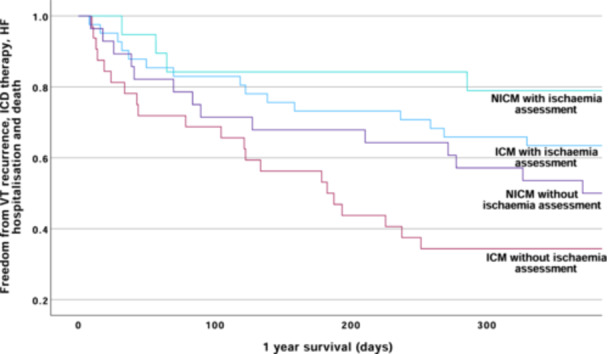
Kaplan–Meier survival curves demonstrating 12‐month event‐free survival comparing those with and without an ischemia assessment by etiology (ICM vs NICM). Amongst those with ICM, 12‐month event‐free survival was 63.4% for those who had an IA versus 34.4% for those who did not, *p* = 0.003). In the NICM cohort, 12‐month event‐free survival was 78.9% for those undergoing IA versus 53.6% for those who did not have an IA, *p* = 0.009.

Within the PS‐matched cohort, those with an ICM who underwent an IA demonstrated greater 12‐month event‐free survival compared to those who did not undergo IA (63.4% vs 34.4%, *p* = 0.003). Similarly, in the NICM cohort, 12‐month event‐free survival was higher in those who underwent IA compared to those who did not have an IA, (78.9% vs 53.6%, *p* = 0.009) (Figure 4).

## 4 Discussion

### 4.1 The Role of Ischemia in Arrhythmogenesis

Managing arrhythmic risk in those with SHD is a key facet of reducing the risk of sudden cardiac death. Early assessment and revascularization of acute coronary ischemia have led to a significant reduction in acute mortality associated with myocardial infarction [7]. Although an association between successful revascularization in the setting of STEMI and reduction in acute arrhythmic events has been proven [8], the longer‐term outcomes for those without acute myocardial infarction are not clear. CAD and cardiac ischemia are known to contribute to the generation of monomorphic VT through several mechanisms. Following a myocardial infarction, healthy myocardium is replaced by a fibrotic scar consisting of a mixture of collagen, extracellular matrix proteins, fibroblasts, other inflammatory cells [9] and, later, infiltrating adipose tissue [10, 11]. Whilst these themselves are not directly arrhythmogenic, the surrounding border zone area harbors surviving bundles of cardiomyocytes interspersed between these nonconducting or slowly conducting areas. This tissue heterogeneity, compounded by altered electrophysiological properties of the surviving myocytes, such as prolongation of the refractory period and reduced excitability [12], can lead to areas of slow conduction, and unidirectional block of the propagating action potential. This forms the basis for re‐entry and ventricular arrhythmias [13, 14].

Ischemic cardiomyocytes undergo characteristic changes to cellular electrophysiology which may increase the risk of ventricular arrhythmia [15]. Intramitochondrial adenosine triphosphate (ATP) levels are depleted, oxidative stress increases, and catecholamines are upregulated [16]. These changes also lead to hibernation and depressed contractile function, which can be ameliorated following reperfusion [17]. Further electrical remodeling at the genetic level alters the expression of major ion channels, manifesting as variations in excitability, conduction velocity, and refractoriness [18], particularly at the borderzone [19, 20].

In NICM, although the distribution and confluence of fibrosis differs from that of ischemic cardiomyopathy, the final common pathway of fibrosis and remodeling is similar [21]. Genetic and acquired conditions which cause NICM lead to similar electrophysiological changes to those seen in ICM, such as reduced conduction velocity and nonuniform tissue anisotropy [22]. Furthermore, “bystander” coronary disease has been found in up to 27% of cases assumed to be pure NICM [23]. These patients with concomitant CAD and underlying NICM have a poorer prognosis than those without CAD [3]. In our cohort, we found a very similar prevalence of significant coronary disease in our NICM cohort (27.5% with moderate or severe lesions on angiography, where performed).

### 4.2 Identification and Reduction of Ischemic Burden

Crucially, we show that on propensity‐score matched analysis, IA was independently associated with a reduction in the adverse events with a 25% absolute risk reduction at 1 year. However, this improved outcome was driven by a reduction in mortality, not by reduction in ventricular arrhythmias or appropriate ICD therapy. These findings contrast, but are not at odds with contemporary evidence in this field. In a recently published cohort study evaluating 97 patients presenting with VT storm, all of whom underwent VT ablation, IA was not shown to lead to any significant mortality improvement [24]. Potential explanations for this difference include the more stable nature of the patients in our cohort (only 7.8% presented with VT storm), higher baseline EF (37.3% vs 30.3% in the VT storm study), and a higher rate of revascularisation—22.3% in our cohort versus 4% in the VT storm study. Furthermore, only 28.1% of patients in our cohort underwent VT ablation within 12 months, compared to 100% in the VT storm study. This reflects the lengthy national waiting times for complex ablation in the United Kingdom at present, with the median time to VT ablation in our cohort of 301 days as well as the lower rates of VT storm. VT ablation is an intervention which will reduce VT recurrence independently to coronary status and thus mitigate any benefit from revascularisation on VT recurrence. The relatively low rate of VT ablation in our cohort serves a strength in that the confounding influence of ablation is reduced. The recent REVIVED‐BCIS2 trial also showed that PCI was not associated with a reduction in mortality or aborted SCD in patients with ICM and severe LV dysfunction [25]. In contrast to our study, less than a quarter of patients in this cohort had experienced a ventricular arrhythmia before intervention, therefore defining themselves as a lower risk group for VT and potentially reducing the observed benefit of IA. Furthermore, revascularisation of significant coronary lesions may be beneficial before considering a patient for VT ablation, to avoid haemodynamic collapse and further ischemic insult during VT induction. Myat et al. reported a significant reduction in the size of ischemic scar on electroanatomical mapping following revascularisation of chronic total occlusion in patients with ischemic VT. This scar modification was observed both acutely after revascularisation as well as on remapping later [26]. Similarly, in our study, those who underwent revascularisation had improved survival on Kaplan–Meier analysis.

A further finding of our study was that coronary disease increased the risk of VT recurrence, ICD therapy, HF hospitalization, and death in a dose‐dependent fashion, with nearly 50% of those with severe coronary disease experiencing an adverse outcome in the following 12 months. There was a nonsignificant trend toward those with ICM having a poorer prognosis than those with NICM. Similar findings have been reported in large‐scale registry data of those with VT [27] and atrial fibrillation [28]. In our cohort, those with ICM were less likely to undergo VT ablation in the 12 months following presentation with VT than those with NICM. We suspect that this is due to the need to undergo revascularisation before VT ablation in the ICM cohort, with nearly one‐third of the ICM cohort undergoing revascularisation. This may delay the time to ablation compared to the NICM cohort.

### 4.3 The Need for Guideline Clarity

Unlike survivors of sudden cardiac death, current clinical guidelines do not have a clear recommendation for the use of coronary imaging in the setting of the first presentation with MMVT and only suggest its consideration when the initial evaluation with ECG and echocardiography are suggestive of coronary disease as the etiology [6, 29]. Furthermore, a previous recommendation in the 2015 ESC guidelines to undertake coronary angiography in those presenting with life‐threatening ventricular arrhythmias was dropped on the subsequent 2022 guidelines [30]. This reflects the lack of high‐quality studies investigating the efficacy in this setting. Our study has demonstrated that regardless of underlying etiology, undergoing IA is associated with an improved prognosis compared to those who do not. IA was of particular benefit to those with ICM. This is likely due to the high prevalence of hemodynamically significant lesions in the ICM cohort who undergo IA. Where IA was performed in the ICM cohort, over two‐thirds of patients were found to have severe coronary disease, and nearly one‐third went on to have coronary intervention. In the nonischemic cohort, over a quarter of patients were found to have hemodynamically significant coronary lesions, almost all of whom went on to have changes to their antithrombotic or heart failure therapy. This emphasizes the frequency of overlapping CAD with NICM and the potential pitfalls of avoiding IA for those with known NICM.

### 4.4 Renal Dysfunction and Arrhythmia

Another significant finding was the association of chronic kidney disease with poorer outcomes. The effect of CKD on cardiovascular mortality is well described and is due to the acceleration of cerebro‐ and cardio‐vascular disease as well as the increased myocardial fibrosis that is seen in those with renal dysfunction [31, 32]. Furthermore, CKD has been shown to increase risk of ventricular arrhythmias, due to shifts in fluid and electrolytes status, altered drug metabolism, the effects of renal replacement therapy as well as increased predisposition to re‐entry via fibrosis [33, 34].

### 4.5 Mechanisms for Improved Outcomes With IA

We propose that the explanation for the improvement in outcomes for those undergoing IA lies in the subsequent treatment optimization which occurs as a direct result. Knowing the coronary status of a patient presenting with VT starts a cascade of treatment including revascularisation, cardiovascular risk modification, and heart failure therapy optimization, which does not take place in those who have not had an IA. We show that those who undergo IA have an increased rate of heart failure therapy optimization (despite similar LVEF between the cohorts) as well as increased anti‐thrombotic therapy. This is a finding which is consistent with findings from the heart failure population undergoing coronary angiography, in both ischemic and non‐ischemic populations [35, 36, 37], but has never previously been documented in the VT population. Having a clearer understanding of a patient's overall cardiovascular pathology allows for optimization of medical therapy, which is known to lead to improved outcomes. Benefit was also seen in the NICM population undergoing IA, despite lack of revascularisation. We postulate that this may be due to the following reasons. Firstly, those with NICM have been shown to die primarily from pump failure, rather than sudden cardiac death [38]. Exclusion of significant coronary disease would allow rapid commencement in optimum heart failure therapy and consequently a reduction in LV remodeling and improvement in pump function. Although this was not assessed in our study, in those with ischemic cardiomyopathy in whom a significant stenosis is found and treated, there may be a delay while the effects of revascularisation are considered before starting heart failure treatment. This delay may be detrimental in some cases. Secondly, the data suggest that for any given heart failure treatment—medical therapy or cardiac resynchronization therapy, the NICM population tends to respond more robustly in terms of improvement in LVEF and reduction in LVEDd [39, 40]. This may be due to higher scar burden in the ICM population, with less inherent reversibility in the substrate compared to those with NICM.

Despite these benefits, it is clearly important to balance any benefits from invasive investigations against potential risk of complication and clearly noninvasive IA has a greater role to play here in the future.

### 4.6 Limitations

This was a single‐center, retrospective study, with the inherent limitations of this study design. Furthermore, the center itself is a specialist center for ventricular arrhythmias, limiting the applicability to the wider population. It is not possible to establish a causal relationship between IA and arrhythmia recurrence due to the nature of the study and possible surrogate clinical markers which were not analyzed. IA and coronary intervention were at the discretion of the senior cardiologist and performed following acquisition of the clinical history and review of relevant investigations such as ECG and echocardiography. This introduces potential selection bias. We feel that this study may form the basis of future prospective randomized studies to evaluate the relationship between IA, revascularization, and VT recurrence. The propensity‐matched nature of the analysis as well as the loss to follow up limited the study size, increasing the chance of a type 1 error. However, we feel that controlling for baseline comorbidities and etiology is of particular importance when trying to evaluate the benefit of an investigation in a relatively unwell population. Furthermore, the findings were very similar on a separate analysis of the non‐PS matched cohort (*n* = 217).

## 5 Conclusions

Patients with SHD presenting with sustained monomorphic ventricular tachycardia who undergo an IA have significantly improved freedom from VT recurrence, appropriate ICD therapies, HF hospitalization, and death compared to those who do not. This is particularly driven by an improvement in all‐cause mortality, but not reduction in arrhythmic events.

## Ethics Statement

This study was granted ethical approval by the local review board and is in compliance with the Declaration of Helsinki. Patients gave their informed consent to participate.

## Supporting information

Supporting information.

## Data Availability

Data are available on request via the corresponding author and where permitted in line with hospital policy.
