# Supplementary material for: The Impact of Coronary Ischemia Assessment on Outcomes in Those With Scar‐Dependent Ventricular Tachycardia
Source: J Cardiovasc Electrophysiol. 2024 Nov 15;36(1):201–11. doi: 10.1111/jce.16495 (PMC11726999; doi:10.1111/jce.16495)
Supplement: Supplementary file 1 — Supporting information. [file JCE-36-201-s001.docx]

# Supplemental data

Supplemental table 1: Baseline characteristics of the propensity-score matched cohort (n = 120) for ischemia assessment. ICM = ischaemic cardiomyopathy, AAD = anti-arrhythmic drug, ARNI = angiotensin receptor-neprilysin Inhibitor, SGLT-2-I = sodium-glucose cotransporter-2 inhibitor.

| **Demographic** | **No Ischemia assessment**  **(n = 60)** | **Ischemia assessment**  **(n = 60)** | **P - value** | **Standardised mean difference** |
| --- | --- | --- | --- | --- |
| Male gender | 51 (85.0%) | 48 (80.0%) | 0.63 | 0.13 |
| Mean age | 68.5 (± 16.9) | 69.1 (± 12.2) | 0.84 | 0.04 |
| ICM | 32 (53.3%) | 41 (68.3%) | 0.13 | 0.31 |
| Hypertension | 19 (31.7%) | 22 (36.7%) | 0.70 | 0.11 |
| Diabetes | 17 (28.3%) | 17 (28.3%) | 1.00 | <0.001 |
| Atrial fibrillation | 23 (38.3%) | 18 (30.0%) | 0.44 | 0.18 |
| Chronic kidney disease | 20 (33.3%) | 21 (35.0%) | 1.0 | 0.04 |
| Baseline EF (%) | 36.4 (± 13.4) | 35.5 (± 12.1) | 0.70 | 0.07 |
| B-blocker | 50 (83.3%) | 46 (76.7%) | 0.50 | 0.17 |
| Amiodarone | 9 (15.0%) | 5 (8.3%) | 0.39 | 0.21 |
| Other AAD | 1 (1.7%) | 1 (1.7%) | 1.0 | <0.001 |
| Antithrombotic | 46 (76.7%) | 47 (78.3%) | 1.0 | 0.04 |
| Statin | 39 (65.0) | 43 (71.7) | 0.56 | 0.14 |
| ACE-I/ARB | 42 (70.0%) | 41 (68.3%) | 1.0 | 0.04 |
| MRA | 24 (40.0) | 19 (31.7%) | 0.50 | 0.17 |
| ARNI/SGLT-2-inhibitor | 4 (6.7%) | 1 (1.7%) | 0.36 | 0.25 |
